# Monitoring of physical health in services for young people at ultra‐high risk of psychosis

**DOI:** 10.1111/eip.12288

**Published:** 2015-10-18

**Authors:** Rebekah Carney, Tim Bradshaw, Alison R. Yung

**Affiliations:** ^1^ Institute of Brain, Behaviour and Mental Health University of Manchester Manchester UK; ^2^ School of Nursing, Midwifery and Social Work University of Manchester Manchester UK

**Keywords:** clinical high risk, lifestyle, psychotic disorders, risk factors, schizophrenia

## Abstract

**Aim:**

People with schizophrenia have poor physical health and high rates of premature mortality. Risk factors for later cardiovascular disease are present from an early stage, and recording of these factors is recommended in first‐episode services. However, it is unclear whether cardiometabolic risk factors are monitored prior to first‐episode psychosis.

**Methods:**

A retrospective analysis was conducted on case notes of individuals accepted into a specialized early detection service for young people at ultra‐high risk for psychosis. Notes were assessed to determine whether the following physical health measures were recorded: height, weight, body mass index, blood pressure, blood glucose and lipids, physical activity levels, smoking status, substance use and alcohol intake.

**Results:**

Forty individuals were deemed at ultra‐high risk for psychosis and accepted into the service. The two measures reported most frequently were whether a person used substances (82.5%) or alcohol (72.5%), but more specific details were not commonly reported. A minority of case files contained information on height (2.5%), weight (7.5%), body mass index (5%), blood glucose (2.5%), smoking status (15%) and physical activity (7.5%). Six case files had no measure of physical health.

**Conclusions:**

Physical health and unhealthy lifestyle factors were not assessed routinely in the specialized service. Clear monitoring guidelines should be developed to establish routine assessment of common metabolic risk factors present in this population.

## Introduction

People with schizophrenia have high rates of physical ill health and premature mortality, with an average life expectancy of 13–30 years lower than the general population.^1^, ^2^, ^3^ The leading cause of death is from chronic physical health conditions, such as cardiovascular disease and diabetes. People with schizophrenia have high rates of cardiometabolic risk factors such as central obesity, hypertension, hyperglycaemia and dyslipidaemia.^4^, ^5^ These cardiometabolic risk factors are associated with side effects of antipsychotic medication^6^, ^7^ and occur soon after starting treatment.^4^, ^8^, ^9^ Additionally, poor cardiovascular health in people with schizophrenia is partly associated with unhealthy lifestyle factors. This includes high rates of alcohol, drug and tobacco use and poor levels of nutrition and low physical activity.^10^, ^11^, ^12^, ^13^, ^14^ These unhealthy lifestyle factors may also be present prior to the initiation antipsychotic medication therapy.^15^, ^16^

Despite available guidelines, rates of physical health screening and assessment in schizophrenia remain poor.^17^ The National Audit of Schizophrenia^18^ revealed only 50% of individuals with schizophrenia had their body mass index (BMI) recorded in secondary care services over 12 months.^19^ As inadequate monitoring and health‐care provision may contribute to poor long‐term outcome, it is important that the disparities in physical health care are addressed.^2^ First‐episode services have developed guidelines emphasizing the importance of capturing cardiometabolic data at regular intervals, starting before individuals begin antipsychotic treatment.^4^ However, it is unclear whether it is necessary and possible to assess and monitor physical health in people before the onset of psychosis, that is, those meeting the ultra high‐risk criteria.

The ultra high risk (UHR) state allows the identification of people in the putative prodrome for psychosis. Criteria have been developed to identify individuals vulnerable to developing a psychotic disorder.^20^, ^21^, ^22^ These have been referred to as the prodromal, ultra‐high risk (UHR), clinical high risk (CHR) and at‐risk mental state (ARMS) criteria.^23^ In order to meet UHR status, a patient must exhibit one or a combination of the following characteristics: presence of attenuated psychotic symptoms, brief limited intermittent psychotic symptoms that spontaneously resolve or a genetic risk combined with a significant recent decline in functioning.^24^ Approximately one‐third of UHR individuals transition to full‐threshold psychotic disorders within 3 years.^25^ These criteria have important implications for early intervention and allow us to examine both the physical and psychological health of young people prior to the development of a first psychotic episode.

Emerging evidence suggests that individuals at UHR for psychosis have high rates of unhealthy lifestyle factors, and poor physical health, prior to the onset of first‐episode psychosis (FEP) (Carney et al.^26^). As the increased rate of cardiovascular disease may be partly due to modifiable risk factors, it seems appropriate to provide adequate monitoring and assessment at this early stage. However, it is unclear whether cardiometabolic risk factors are monitored effectively by UHR services. We therefore aimed to (i) review case notes of UHR individuals to see if cardiometabolic risk factors are recorded and (ii) assess the physical health of UHR individuals from the information available. Case notes were taken from a 12‐month intake of referrals into a specialized UHR early detection service.

## Method

A retrospective analysis was conducted on the case notes of clients accepted into a specialized early detection and intervention service based in Greater Manchester, UK.

### Sample

The study population consisted of a 12‐month intake into the Early Detection and Intervention Team (EDIT) between October 2013 and October 2014. EDIT is based in a UK National Health Service primary care setting in Salford and Wigan and accepts referrals of people aged between 14 and 35 who are at UHR for developing psychosis, defined according to the Comprehensive Assessment of At‐Risk Mental States.^27^

### Outcome measures

A structured audit tool was used to assess whether the following physical health measures were recorded: height, weight, body mass index (BMI), blood pressure, blood glucose, blood lipids, physical activity levels, smoking status and substance use and alcohol intake. To maintain patient confidentiality, no personal identifiable information was extracted. Demographic information consisted of age at time of acceptance, location of service and whether an individual transitioned to psychosis. For each individual measure, we noted (i) whether the variable had been assessed and (ii), if so, relevant information was recorded. If no reference to the variable was made, it was assumed that it had not been assessed. For example, if tobacco or alcohol use was measured, the frequency and quantity of use was recorded. For substance use, the name of substance was also recorded, in addition to any previous drug use. If information on physical activity were available, the type of exercise and total time spent per week was extracted.

### Statistical analysis

Data analysis was conducted in SPSS version 22.0 (IBM Corp., Armonk, NY, USA).^28^

## Results

**Figure 1:**
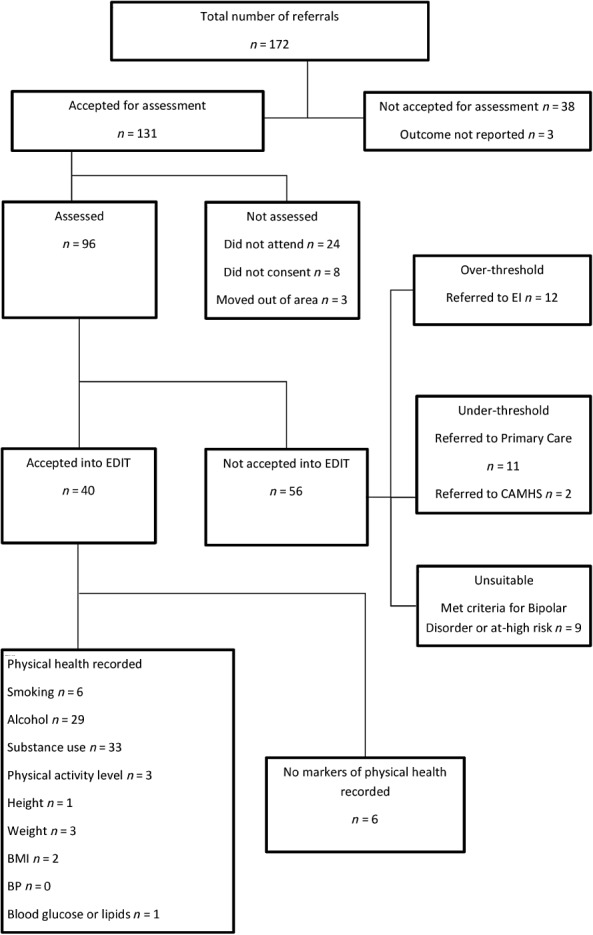
Flow chart of referrals.

A total of 131 referrals were accepted for assessment by EDIT (Fig. 1). Of these 131, 40 individuals met the UHR criteria and were accepted into the clinical service. Case notes of these individuals were reviewed using a structured audit tool created by the researchers. Ninety‐one referrals were not accepted as they were under‐threshold (*n* = 35), met criteria for first‐episode psychosis (*n* = 12), met criteria for bipolar or bipolar at risk (*n* = 9) or moved out of the area (*n* = 3). A further 32 were not assessed due to failure to attend appointments (*n* = 24), or failure to provide consent (*n* = 8).

### Demographic data

The mean age at time of acceptance to EDIT was 21.4 (standard deviation (SD) = 5.5, range 14–35). Slightly more service users came from the Wigan site (*n* = 23, 57.5%) than Salford (*n* = 17, 42.5%). A small percentage of case files contained information on transition to psychosis after being referred to EDIT (*n* = 3, 7.5%).

### Information recorded

The physical health of UHR individuals was not assessed routinely (see Table 1). None of the files recorded physical health data for all factors. In total, six (15%) of the individuals accepted into the EDIT service over the previous 12 months did not have any documentation of physical health measures or information on unhealthy lifestyle factors. Anthropometric variables were reported in a minority of case files: height (*n* = 1, 2.5%), weight (*n* = 3, 7.5%), BMI (*n* = 2, 5%). Blood pressure was not documented for any referral accepted into EDIT, and blood glucose and lipid testing was provided for only one individual. Physical activity levels were not routinely assessed, as reference to physical activity was only made in three cases (7.5%).

**Table 1:** The proportion of case notes containing physical health information

Factor	Physical health measures noted, *n* (%), (*n* = 40)
Height	1 (2.5%)
Weight	3 (7.5%)
Body mass index	2 (5%)
Blood pressure	0 (0%)
Blood glucose/lipids	1 (2.5%)
Smoking status	6 (15%)
Alcohol use	29 (72.5%)
Substance use	33 (82.5%)
Physical activity	3 (7.5%)

Current alcohol use was measured in 29 cases (72.5%). Approximately half of these cases also recorded alcohol frequency (*n* = 15, 51.7%) (Table 2). Quantity of alcohol consumed was also recorded in 12 cases (41.2%). The majority of case files contained a reference to substance use (*n* = 32, 82.5%). In these cases, the frequency of substance use was reported more often than the quantity (*n* = 28, 87.5%; *n* = 25, 78.1%). Smoking status was only available in a minority of cases (*n* = 6, 15%). From the six case files that reported smoking status, five contained information on frequency and quantity of tobacco use (*n* = 5, 83.3%).

**Table 2:** Proportion of people reporting tobacco, alcohol or substance use

Factor	Additional measures recorded *n* (%)
Smoking recorded (*n* = 6)	
Smoking frequency	5 (83.3%)
Smoking quantity	5 (83.3%)
Alcohol use recorded (*n* = 29)	
Alcohol frequency	15 (51.7%)
Alcohol quantity	12 (41.2%)
Substance use recorded (*n* = 32)	
Substance frequency	28 (87.5%)
Substance quantity	25 (78.1%)

### Physical health data

### Anthropometric measurements

Height was reported for one individual (180 cm), and weight was recorded in three cases (mean = 52.38 kg, SD 16.26, range 34.92–67.10 kg). BMI values were available in two instances (m = 18.75, SD 2.62, range 16.9–20.6).

### Alcohol use

From the information available, the majority of people used alcohol (*n* = 24, 82.8%). Alcohol abuse or misuse was documented for a high percentage of alcohol users (*n* = 9, 37.5%). For people who reported alcohol use, some case notes contained information about the specific drinks consumed, for example, one bottle of wine per week. This was then converted using the NHS unit calculator to provide the best estimate of alcoholic units per week. The average weekly intake of alcoholic units was 96.03 (SD 60.64, range 8–186). Nine referrals disclosed how frequently they consumed alcohol. The highest proportion of people used alcohol daily (*n* = 4), followed by 1–2 days per month (*n* = 2), 3–5 days (*n* = 1), 6–9 days (*n* = 1) and 10–19 days per month (*n* = 1).

### Substance use

A high percentage of individuals reported no current drug use (*n* = 21, 65.6%). The remaining data reported drug use (*n* = 8, 25%), abuse (*n* = 2, 6.3%) or dependence (*n* = 1, 3.1%). Where applicable the substance name was reported in all but one case (*n* = 10, 90.9%). Almost all substance users used cannabis (*n* = 8, 72.7%); other substances included cocaine, heroin, ketamine and legal highs. Lifetime or previous substance use was reported in eight cases. The frequency and quantity of substance use was difficult to quantify, as it was often reported in terms of how much a person spent on the substance per week, for example, £100 per week. However, from the limited information available, it appears that most people who used substances did so on a daily basis (*n* = 5).

### Smoking

From the limited information available, 50% (*n* = 3) of service users smoked. UHR smokers smoked daily (*n* = 2) and the amount ranged from two to five cigarettes per day (*n* = 1) to 6 to 10 per day (*n* = 1).

### Physical activity

When physical activity was reported, all three stated the type of activity: daily jogging (*n* = 1), pole fitness (*n* = 1) and yoga (*n* = 1). One case stated classes were attended twice per week averaging 120 min of exercise.

## Discussion

Physical health monitoring and assessment in this specialized UHR service is low. None of the referrals had a complete documented assessment of physical health upon intake to the service, and six case files contained no measure of physical health. Anthropometric assessments were substantially lacking within case notes, and blood glucose assessments were found for only one individual. Although it was often reported whether a person used alcohol, substances or tobacco, more specific details relating to the extent of use were not recorded.

Because of the limited data available from the case notes, we are unable to assess the physical health and proportion of lifestyle risk factors with any certainty. However, in a large proportion of cases specific details on alcohol and substance use were only documented when it was of concern to the clinician or client. This is reflected in the large amount of average units per week provided for alcohol use. The most commonly reported drug used was cannabis. This is consistent with the findings of a previous review that also found the most commonly reported drug in UHR individuals was cannabis, and the rates of cannabis use were much higher than found in the general population (Carney et al.^26^).

The findings from this service evaluation are similar to the national audit conducted by Crawford et al.,^19^ which found that assessment and treatment of physical health complaints in severe mental illness are below recommended standards. This is an important failure of care considering that a large proportion of premature mortality and morbidity seen in these patients may be preventable.^29^, ^30^ Early monitoring of physical health is possible through UHR services; however, at least in this service in Greater Manchester it does not occur.

Barriers to providing effective management and assessment of physical illness in mental health services have previously been discussed.^2^, ^31^ A key issue is a lack of clarity and consensus regarding with whom the responsibility lies.^2^, ^31^, ^32^, ^33^ Additionally, the lack of integration between mental and physical health services contributes to suboptimal health care. In many cases, people with schizophrenia only access health care via mental health services, suggesting a more holistic approach to service provision is required. Other factors reported by mental health outreach clinicians include uncertainty over what should be monitored and when, lack of confidence interpreting abnormal results and limited access to equipment.^31^, ^34^ A large proportion of mental health professionals fail to discuss metabolic side effects of medication due to issues around non‐adherence.^34^ Although this does not usually apply to the UHR group, it is an important reflection of how motivations of the clinician may affect screening in mental health care.

### Clinical implications and recommendations

This audit provides evidence that improvements are needed for physical health‐care provision in UHR. NICE guidelines currently recommend routine monitoring of weight, cardiovascular and metabolic parameters for people with psychosis and schizophrenia.^35^ The implementation of these recommendations in early intervention services is a national priority. A leading C‐QUIN target for 2014/2015 focuses on improving physical health care for people with psychosis to reduce premature mortality. Local trusts are paid for routine monitoring and assessment of cardiometabolic parameters, including lifestyle information, BMI, blood pressure, glucose regulation and blood lipids.^36^

Despite substantial national targets for psychosis, the same recommendations are not given for at‐risk populations. This is concerning, as UHR individuals display a wide range of risk factors for future ill health, which are largely preventable. Therefore, clear monitoring guidelines are required within early detection services to encourage screening of early risk factors. One way this could be implemented is to use a concise physical health assessment tool, administered at, or soon after intake to the early detection service. The Health Improvement Profile was a tool designed to aid mental health nurses with physical assessments in people with severe mental illness.^37^, ^38^ A similar approach could be used in the UHR group.

It is important to gather metabolic information during the UHR phase, as individuals are usually antipsychotic naïve, thus allowing baseline cardiometabolic risk to be assessed. Therefore, if later transition to psychosis occurs, metabolic indices can be compared to see whether any abnormalities that may be present are an inherent part of the illness progression or a result of later antipsychotic medication.

### Strengths and limitations

This service review is the first to be conducted within a specialized early detection and intervention setting. The findings carry important implications for future service development and provide a baseline to improve upon. However, the small sample size and cross‐sectional design are limitations. As this assessment was restricted to one UK service in Greater Manchester, the findings may not be representative of other early detection services. However, a lack of existing literature on the topic indicates that physical health may not have been assessed routinely across the majority of UHR services. The large proportion of missing information meant no statistical analysis could be conducted on the sample to assess markers of physical health.

## Conclusions

Physical health was not assessed routinely in the EDIT service. Clear monitoring guidelines should be developed to establish routine assessment of common metabolic risk factors present in this population. Appropriate interventions can then be targeted to prevent future ill health.
